# Yeast's balancing act between ethanol and glycerol production in low‐alcohol wines

**DOI:** 10.1111/1751-7915.12488

**Published:** 2017-01-13

**Authors:** Hugh D. Goold, Heinrich Kroukamp, Thomas C. Williams, Ian T. Paulsen, Cristian Varela, Isak S. Pretorius

**Affiliations:** ^1^ Department of Chemistry and Biomolecular SciencesMacquarie UniversitySydneyNSW2109Australia; ^2^ New South Wales Department of Primary IndustriesLocked Bag 21OrangeNSW2800Australia; ^3^ The Australian Wine Research InstitutePO Box 197AdelaideSA5064Australia

## Abstract

Alcohol is fundamental to the character of wine, yet too much can put a wine off‐balance. A wine is regarded to be well balanced if its alcoholic strength, acidity, sweetness, fruitiness and tannin structure complement each other so that no single component dominates on the palate. Balancing a wine's positive fruit flavours with the optimal absolute and relative concentration of alcohol can be surprisingly difficult. Over the past three decades, consumers have increasingly demanded wine with richer and riper fruit flavour profiles. In response, grape and wine producers have extended harvest times to increase grape maturity and enhance the degree of fruit flavours and colour intensity. However, a higher degree of grape maturity results in increased grape sugar concentration, which in turn results in wines with elevated alcohol concentration. On average, the alcohol strength of red wines from many warm wine‐producing regions globally rose by about 2% (v/v) during this period. Notwithstanding that many of these ‘full‐bodied, fruit‐forward’ wines are well balanced and sought after, there is also a significant consumer market segment that seeks lighter styles with less ethanol‐derived ‘hotness’ on the palate. Consumer‐focussed wine producers are developing and implementing several strategies in the vineyard and winery to reduce the alcohol concentration in wines produced from well‐ripened grapes. In this context, *Saccharomyces cerevisiae* wine yeasts have proven to be a pivotal strategy to reduce ethanol formation during the fermentation of grape musts with high sugar content (> 240 g l^−1^). One of the approaches has been to develop ‘low‐alcohol’ yeast strains which work by redirecting their carbon metabolism away from ethanol production to other metabolites, such as glycerol. This article reviews the current challenges of producing glycerol at the expense of ethanol. It also casts new light on yeast strain development programmes which, bolstered by synthetic genomics, could potentially overcome these challenges.

## Today's sunshine is tomorrow's wine

Wine's history parallels that of the civilization of humankind. For more than 7000 years, humans have exploited the fermentation power of yeast as a means of preservation of grape juice (Pretorius, 2000). We will never know who tasted wine for the very first time. However, we do know that the pleasant taste and ‘magical’ psychotropic side‐effects of the preservative agent ‒ alcohol ‒ in spontaneously fermenting damaged grapes convinced the early tipplers around the Black and Caspian Seas to keep practicing and refining their newly discovered invention of winemaking from one harvest to the next.

**Figure 1:**
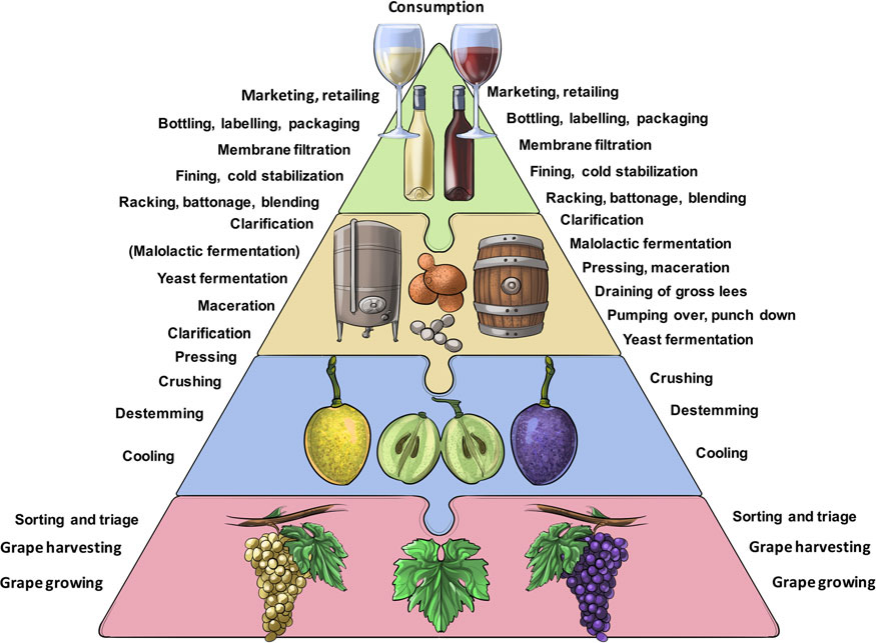
A schematic outline of the sequence of the main steps in the production of white wine (left) and red wine (right). The world's annual production of almost 30 billion litres of wine from approximately 8 million hectares of vineyards is made roughly following the same production procedures in the vineyard and winery. Obviously, production steps to optimally manage vineyards differ to suit specific geographic locations and grape variety. Details in the sequential steps of winemaking also vary with wine type and style (white, red, sweet, fortified and sparkling wine). Generally, the first step entails the crushing of grapes to liberate the sugar in the juice for fermentation. Fermentation can occur spontaneously or after inoculation of the grape must with one or more specific yeast strains (e.g. different strains of *Saccharomyces cerevisiae* in single‐species ferments or in combination with other non‐*Saccharomyces* species – such as *Torulaspora delbrueckii* – in mixed ferments (Jolly *et al*., 2014). The pre‐ and post‐fermentation treatments of grape must (e.g. clarification and stabilization) also vary depending on wine type and wine style. Following the yeast‐driven alcoholic fermentation during red winemaking, a secondary bacterial fermentation – malolactic fermentation – is facilitated by lactic acid bacteria of which *Oenococcus oeni* is the best known species. Malolactic fermentation is also used in some white wine styles. To reproducibly produce predetermined wine styles according to preferences of targeted segments of consumer markets, winemakers make multiple choices across the entire value chain. These choices include the use of fermenters (e.g. stainless steel tanks, oak barrels), enzyme treatments, oak maturation, certain types of packaging materials (e.g. cork bottles closures, screw caps) and marketing strategies.

Throughout the early ages, the ancients argued that *today's sunshine is tomorrow's wine*. In the words of Galileo Galilei, wine became known as *sunlight, held together by water*. In many cultures, a *meal without wine was like a day without sunshine*. With every vintage came new quirky traditions and incremental innovations. It was only at the end of the 19th century when famed scientist Louis Pasteur determined the role of living yeast cells in the conversion of sugary grape must into wine, thereby turning the ‘practical art’ of winemaking into an applied science (Liti, 2015). Since then, detailed knowledge of yeast's fermentative metabolism – alongside the development of modern vineyard practices, winemaking equipment and packaging material, as well as ever‐changing consumer preferences – placed the global wine industry on a never‐ending cyclical journey of *today's innovation is tomorrow's tradition* across the entire *from‐grapes‐to‐glass* value‐chain (Fig. 1).

One such consumer‐driven *innovation* that has become a *tradition* over the past three decades is the extension of the time before grapes are harvested in dry, warm wine‐producing regions of the world. As more consumers responded favourably to richer and fruitier styles of wine, vintners increased the so‐called hang time of grapes. These later‐harvested grapes produced wines not only with enhanced ripe fruit flavours and wine colour intensity, but also with reduced undesirable unripe, vegetal wine flavours (Varela *et al*., 2015). However, riper grapes have higher sugar concentrations (> 240 g l^−1^) which result in higher alcohol concentration (> 13.5% v/v) in the final wine. Rich, ripe fruit flavours and more intense colour but higher alcohol is the ‘double‐edged sword’ of this wine style category, which is often described as ‘bottled sunshine’. The challenge of this conundrum is whether winemakers could keep bottling the highly desirable ‘sunshine flavours’ without the risk of excessive alcohol concentrations in their wines.

The alcoholic strength of table wine usually ranges between 9% and 15% (v/v) with the great majority between 11.5% and 13.5% (v/v). However, in sunny, warmer regions, the average alcohol content has risen by approximately 2% (v/v) over the past 30 years or so. Where it used to be rare to encounter wines with alcohol concentrations of more than 14% (v/v) before the 1980s, it is now not uncommon to see wines with an alcohol concentration of higher than 16% (v/v) (Varela *et al*., 2015).

**Figure 2:**
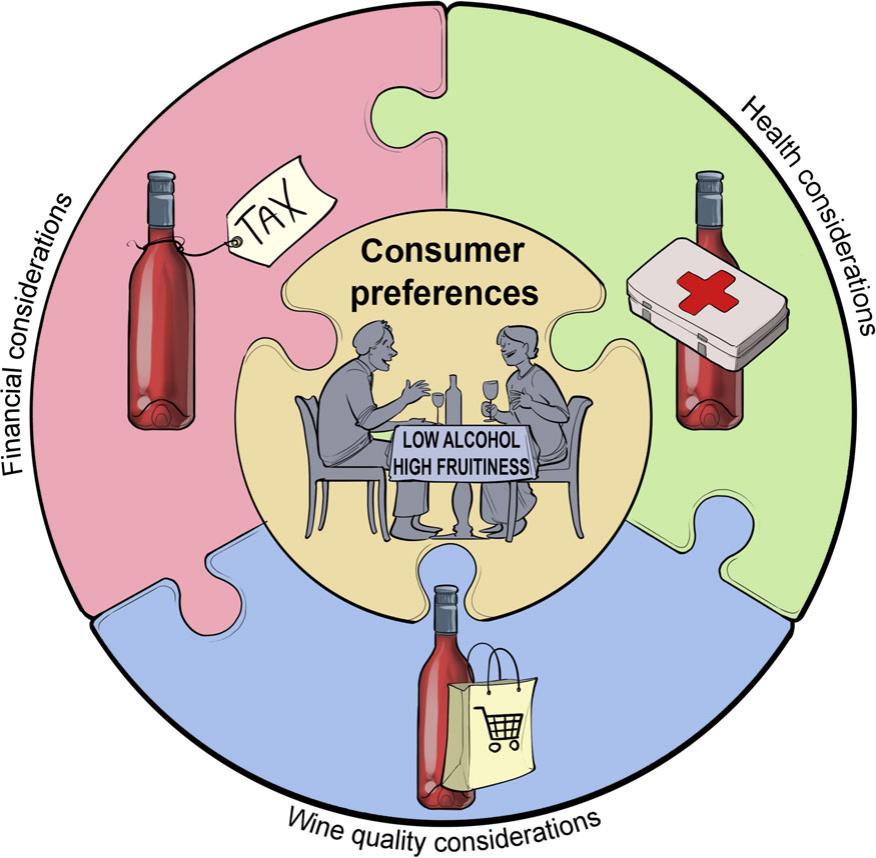
A schematic representation of the main drivers behind the demand for wines containing lower concentrations of alcohol. Excessive concentration of alcohol in wine can have several important implications relating to wine quality, financial and health considerations. Too much alcohol in certain wine styles can compromise the overall quality of the wine by masking the aroma and flavour and increasing the perception of ‘hotness’, viscosity and/or astringency on the palate and making the wine appear unbalanced. Costs to purchasers and consumers are higher in countries where duties are levied according to alcohol content. Despite a growing body of evidence indicating the health benefits of responsible, light‐to‐moderate wine consumption compared with other alcoholic beverages, wine continues to be caught up in the public discussion of the negative social, medical and economic impacts of alcohol abuse.

There are three main interconnected drivers that explain the interest of the global wine industry in taking control of alcohol concentration in wine – these relate to economic, health and quality issues (Fig. 2). First, there are countries that apply financial imposts on wine with a ‘high’ alcohol concentration. This increases the end cost of the wine to the purchaser and the consumer in those countries. Second, in today's increasingly health‐ and safety‐conscious society, wines with high alcohol concentration attract constant negative commentary from health professionals, lawmakers, media, anti‐alcohol advocacy groups and politicians. The harmful effects of excessive alcohol consumption on communities and the concomitant burden on health care, law enforcement services and economic productivity have been widely reported. Third, too much alcohol in wine can negatively affect the sensory properties of a wine. Although many wines with higher alcohol concentration are full‐bodied and rich in ripe fruit flavours, in some cases and depending on wine style, too high a concentration of alcohol can be perceived as a ‘hotness’ on the palate, making overly alcoholic wines appear unbalanced. In terms of overall wine quality, balance between alcohol strength, acidity, tannin, sweetness and fruit flavour intensity is extremely important.

**Figure 3:**
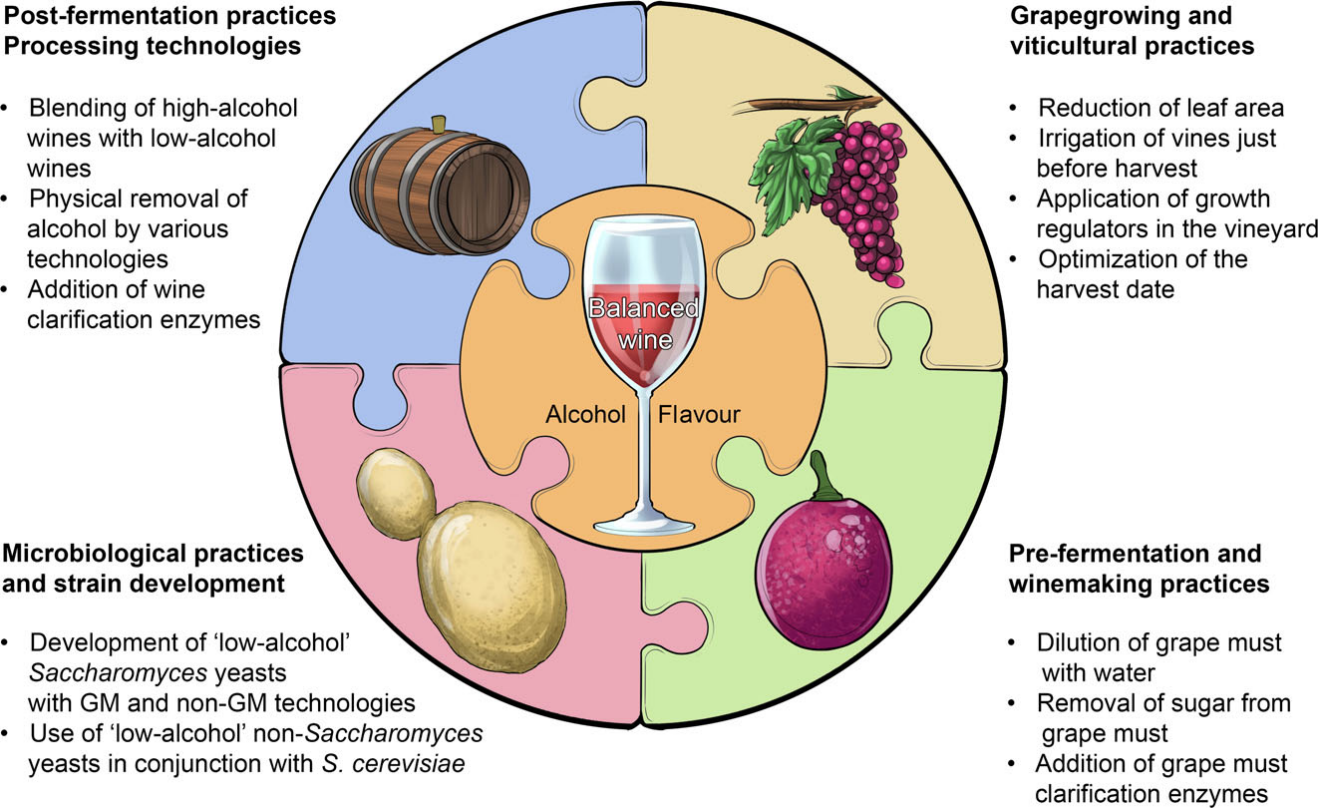
A schematic representation of the main strategies across the value chain for decreasing the concentration of alcohol in wine. These strategies focus on (i) grape growing and viticultural practices; (ii) pre‐fermentation and winemaking practices; (iii) microbiological practices and strain development programmes; and (iv) post‐fermentation practices and processing technologies. Viticultural practices, such as reducing the *leaf‐area‐to‐fruit‐mass* (LA:FM) ratio (which lowers the sugar concentration in grape berries) and harvesting grapes earlier when grapes have lower sugar concentrations, will both result in wines with less alcohol. In some instances, irrigation before harvest and the exogenous application of growth regulators to either the bunch zone or the whole canopy might also delay sugar ripening, thereby resulting in juices with lower sugar content. Pre‐fermentation and winemaking practices include the dilution and blending of high‐sugar grape juice with juice from early harvested low‐sugar grapes or the treatment of grape must to remove glucose and fructose (e.g. the use of nanofiltration to concentrate and remove sugar from grape must or the addition of glucose oxidase preparations that converts glucose into gluconic acid). Microbiological strategies are largely focussed on the development of yeast strains with decreased efficiencies of ethanol production (e.g. strains that produce higher concentrations of glycerol at the expense of ethanol). Post‐fermentation practices and processing technologies include blending high‐alcohol wine with low‐alcohol wine or the physical removal of alcohol following fermentation (e.g. membrane‐based systems such as reverse osmosis, evaporative perstraction, pervaporation, and osmotic distillation, vacuum distillation, spinning cone technology and supercritical CO_2_ extraction).

These three drivers and a growing market demand are calling for a reduction of alcohol concentration in wines, preferably without compromising wine flavour, consumer acceptance or increasing the cost of production (Varela *et al*., 2015). Researchers are focussing on four main strategies across the production chain to reduce alcohol concentration in wine (Fig. 3). Strategies in the vineyard are focussed on (i) decreasing the leaf‐area‐to‐fruit‐mass ratio in an attempt to curtail photosynthesis and sugar accumulation in grapes; (ii) applying growth regulators to either the bunch zone or whole vine canopy as a means to delay sugar ripening; and (iii) optimizing the harvest date by not harvesting overly ripe grapes with excessive sugar concentration. Strategies aimed at pre‐fermentation and winemaking practices focus on (i) blending early harvested, low‐sugar grapes with well‐ripened, flavour intense grapes; (ii) limited dilution of grape must with water; and (iii) removal of sugar from grape must via nanofiltration and the addition of enzymes such as glucose oxidase from *Aspergillus niger*. Post‐fermentation and processing technologies that could be used include (i) the blending of high‐alcohol wine with low‐alcohol wine; (ii) the physical removal of alcohol by using membrane systems (e.g. reverse osmosis, evaporative perstraction and pervaporation), osmotic distillation, vacuum distillation, spinning cone technology and supercritical carbon dioxide extraction. The advantages, disadvantages and effectiveness of these viticultural, pre‐fermentation and post‐fermentation strategies are still being debated (recently reviewed by Varela *et al*., 2015). This article appraises the fourth winemaking strategy, which is aimed at microbiological practices and yeast strain development programmes.

## Not all yeasts are created equal under the sun

There are roughly 150 described yeast genera and of the 1500 known yeast species more than 40 have been found in vineyards and wineries around the world (reviewed by Jolly *et al*., 2014). The surface of unripe grape berries presents nutrient limitations for microbial growth; however, that situation changes as the berries ripen and/or are damaged. The number and diversity of yeasts on cellar surfaces in wineries are highly dependent on cellar hygiene practices. Grape must presents a rich nutritive environment for yeasts, but factors such as low pH, high osmotic pressure, low water activity and the presence of sulfite restrict several yeast species that would otherwise flourish (Pretorius, 2000; Delfini and Formica, 2001).

In spontaneous wine fermentation, a diverse range of indigenous non‐*Saccharomyces* yeasts participate in a progressive pattern during the early phases of the fermentation process until the ethanol concentration reaches 3–4% (v/v); after that, *Saccharomyces* yeasts dominate the fermentation process. The final stages of fermentation are invariably dominated by alcohol‐tolerant strains of *Saccharomyces cerevisiae* (Cray *et al*., 2013). In inoculated ferments, *S. cerevisiae* is universally preferred for initiating the fermentation process (Jolly *et al*., 2014) and its primary role is to catalyse the rapid, complete and efficient conversion of grape sugars to ethanol, carbon dioxide and other minor, but important metabolites without the development of off‐flavours (Pretorius, 2000; Borneman *et al*., 2007).

Over seven millennia, wine strains of *S. cerevisiae* co‐evolved with winemaking practices. *S. cerevisiae* has developed a so‐called Crabtree‐positive carbon metabolism as a highly efficient strategy for sugar utilization (with a preference for glucose over fructose) that maximizes ethanol production (Pfeiffer and Morley, 2014). This adaptation enables energy generation under fermentative or anaerobic conditions and restricts the growth of competing microorganisms (including non‐*Saccharomyces* yeasts) by producing toxic metabolites, such as ethanol and carbon dioxide (Varela *et al*., 2012). In this potentially toxic environment, any non‐genetic strategy aimed at reducing the alcohol concentration in wine will therefore have to include practical ways of giving less efficient *Saccharomyces* and non‐*Saccharomyces* yeasts a head start during fermentation to convert some of the sugar in grape must to metabolites other than ethanol before the highly efficient *S. cerevisiae* strains become dominant.

**Figure 4:**
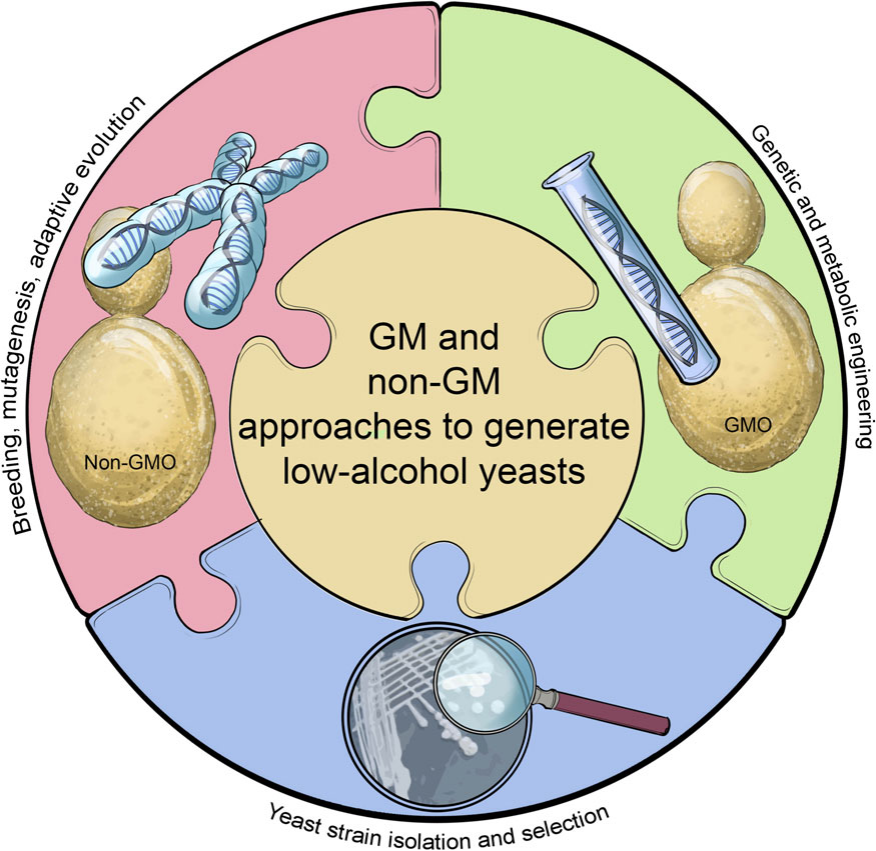
A schematic representation of approaches to generate low‐alcohol wine yeast strains includes strain selection and strain development. Some techniques alter limited regions of the genome, whereas other techniques are used to recombine or rearrange the entire genome. The most common techniques include strain isolation, selection of variants, mutagenesis and hybridization (mating, rare‐mating and intraspecies spheroplast fusion). Strains derived from these approaches are all considered as non‐genetically modified organisms (non‐GMOs) and are being used in commercial winemaking. The use of genetic engineering, metabolic engineering and genome engineering offers precise and very powerful ways to alter specific characteristics of wine yeasts; however, strains resulting from such approaches are GMOs and currently cannot be used for commercial winemaking in most countries. These GM strains do, however, offer invaluable advantages in terms of gaining insights into the fundamentals of what makes a high‐performing wine yeast tick.

Microbial approaches to curb the production of ethanol during wine fermentation include (i) the isolation of new low‐alcohol *Saccharomyces* and non‐*Saccharomyces* yeasts with sound oenological properties; (ii) the use of adaptive evolution (also known as directed evolution) to develop low‐alcohol variants of existing wine strains of *S. cerevisiae*; and (iii) the application of genetic modification (GM) techniques to enable the redirection of sugar carbon away from ethanol to other end‐points such as glycerol (Fig.**** 4).

## Serving up non‐*Saccharomyces* yeast as an entrée to alcoholic fermentation

Spontaneous fermentation is a traditional winemaking practice which exploits the endogenous yeasts (and bacteria) from a particular vineyard and winery to ferment grape juice into a wine rather than fermentation using single strains of *S. cerevisiae* (Díaz *et al*., 2013). From a commercial standpoint, spontaneous fermentation is accompanied with significant risks: with irreproducibility from one vintage to another, stuck fermentations, undesirable flavours and poor wine qualities being some of the frequent problems associated with this practice (Jolly *et al*., 2014). Under ideal winemaking conditions, a variety of non‐*Saccharomyces* yeasts flourish at the start of the fermentation, but are quickly outcompeted by natural *S. cerevisiae* strains, due to their tolerance to the initial high sugar concentrations, sulfite additions and the high ethanol concentration that accumulates towards the end of the fermentation. A fine balance between these population successions is needed to obtain the desired results: if the *S. cerevisiae* succession is too slow, it might result in stuck fermentations and if it is too fast, the wine might lack aromatic complexity. This unpredictability is also common to co‐inoculation of different yeast species. As such, a low impact of non‐*Saccharomyces* yeasts on the aroma complexity of wine is usually due to the rapid succession of *S. cerevisiae* in the fermentation (Bellon *et al*., 2011). For these reasons, even though single inocula strategies limit the sensory complexity and rounded palate, most winemakers prefer maintaining robustness and stability by pitching grape juice with well‐characterized wine strains of *S. cerevisiae*.

Generally, in spontaneously fermenting grape must that is not seeded with a high‐density inoculum of *S. cerevisiae*, there is a sequential succession of non‐*Saccharomyces* species of *Candida*,* Cryptococcus*,* Hanseniaspora* (*Kloeckera*), *Metschnikowia*,* Pichia* and *Rhodotorula* (Jolly *et al*., 2014). The contribution of these yeasts' metabolites to wine flavour depends on how active they are during the initial phases of fermentation, and this in turn depends on how well, and for how long, they can cope with the high osmotic pressure, equimolar mixture of glucose and fructose, high sulfite concentration, suboptimal growth temperature, decreasing nutrients as well as increased alcohol concentrations and anaerobic conditions.

There is a growing interest to deliberately co‐inoculate grape must with non‐*Saccharomyces* species (e.g. *Hanseniaspora uvarum*,* Lachancea thermotolerans*,* Metschnikowia pulcherrima*,* Pichia kluyveri*,* Schizosaccharomyces malidevorans*,* Starmerella bacillaris*,* Torulaspora delbrueckii*, and *Zygosaccharomyces bailii*) with one or more wine strains of *S. cerevisiae*. It is believed that the participation of these selected non‐*Saccharomyces* yeasts in the initial phases of wine fermentation would enrich the flavour profiles and complexity of the wine and, in some instances, convert some of the grape sugars to metabolites other than ethanol.

Indeed, several non‐*Saccharomyces* species have shown potential for producing reduced‐alcohol wines when used as single inocula or in mixed inoculation regimes with *S. cerevisiae*. For example, a selected strain of *M. pulcherrima* was successfully used to produce Chardonnay and Shiraz wines with 0.9% and 1.6% (v/v) less ethanol, respectively, than control wines produced with *S. cerevisiae* (Contreras *et al*., 2014). Similarly, strains of the species *H. uvarum*,* Zygosaccharomyces sapae*,* Z. bailii* and *Zygosaccharomyces bisporus* were identified as candidates with the potential to produce wines with reduced ethanol concentration when used as single inocula in Verdicchio and Trebbiano musts (Gobbi *et al*., 2014). In another study, *Hanseniaspora opuntiae* and *H. uvarum* strains were reported to be able to produce Sauvignon Blanc and Pinotage wines with lower ethanol concentration than *S. cerevisiae* wines (Rossouw and Bauer, 2016). Strains of the species *S. bacillaris* have also been used to produce reduced‐alcohol wines; Barbera wines fermented sequentially with *S. bacillaris/S. cerevisiae* showed 0.7% (v/v) lower ethanol concentration than *S. cerevisiae* wines in 200‐l industry trials (Englezos *et al*., 2016).

There is obvious merit in further pursuing research into ‘multispecies’ ferments because the concept of ‘mixed fermentation’ is not new to the wine industry. Commercial mixtures of *T. delbrueckii* or *Kluyveromyces* (now *Lachancea*) *thermotolerans* in conjunction with *S. cerevisiae* are already being used to produce wines with richer and rounder flavours and, in some cases, fruity notes (Jolly *et al*., 2014). Although these commercialized flavour‐enhancing yeast blends were not primarily developed to reduce the concentration of ethanol in wine, other non‐*Saccharomyces* yeasts, such as carefully selected strains of the yeast species mentioned above, could be developed as co‐cultures for the reduction of alcohol concentration in wine. The choice and compatibility of such non‐*Saccharomyces* and *S. cerevisiae* low‐alcohol companions will be crucial and dependent on wine type.

## Directing yeast metabolism away from ethanol production

Sugar fermentation in *S. cerevisiae* is a redox neutral process influenced by the NAD^+^/NADH balance. Most of NAD^+^ is reduced during glycolysis in the reaction catalysed by the enzyme glyceraldehyde‐3‐phosphate dehydrogenase. For glycolysis to proceed, it is essential to recycle NAD^+^ and oxidize NADH, otherwise glycolytic flux decreases, potentially leading to the depletion of ATP energy charge which could be lethal for the cell (Verduyn *et al*., 1990). Most of the NADH produced during glycolysis is subsequently oxidized during ethanol formation, although NAD^+^ regeneration can also occur via the cytosolic production of glycerol which is catalysed by the enzyme glycerol‐3‐phosphate dehydrogenase (Kutyna *et al*., 2010). In addition to ethanol, the production of several metabolites that can influence wine flavour and aroma, such as glycerol and acetic acid, is linked to redox balance. Thus, altering NAD^+^/NADH balance has been used to redirect carbon flux towards desired end‐points, for example glycerol overproduction, and away from ethanol formation.

Several metabolites can alter redox balance and/or influence yeast metabolism and therefore decrease ethanol production, and these include furfural, vanillin, glycolaldehyde, some organic acids such as cinnamic acid, benzoic acid, formic acid and propionic acid, sodium or potassium chloride, sulfur dioxide and sodium carbonate (Kutyna *et al*., 2010; Vejarano *et al*., 2013). Although some of these metabolites can be added to fermenting must, they might affect wine sensory profile. In addition, some conditions, such as increasing fermentation temperature, can also alter yeast metabolism and divert carbon away from ethanol production. It is very likely, however, that such conditions will affect dramatically wine flavour and sensory profile. Considering that the implementation of the strategies mentioned above can be incompatible with wine production, research efforts have focused on employing such strategies to develop yeast strains with particular metabolic traits, for example glycerol overproduction.

## Breeding of *Saccharomyces* hybrids for increased glycerol and reduced ethanol production

Mutagenesis and genetic breeding practices have been used quite successfully to develop *S. cerevisiae* wine strains to improve specific traits (e.g. robustness, fermentation performance and sensory attributes) better suited for certain winemaking practices and wine styles (reviewed by Pretorius, 2000). For example, several low‐H_2_S‐producing mutants of widely used *S. cerevisiae* wine strains have been developed and successfully commercialized under the names *Advantage*,* Platinum* and *Distinction* (Cordente *et al*., 2009; Pretorius *et al*., 2012). Intraspecies hybridization (mating of *S. cerevisiae* haploids of opposite mating types to yield heterozygous diploids) has also been used effectively to breed commercial wine yeasts (e.g. VIN13 and VL3) with superior winemaking properties tailored for certain wine styles (Van der Westhuizen and Pretorius, 1992; Pretorius, 2000).

Recently, it has been discovered that many wine (and brewing) yeast strains are in fact interspecific hybrids of *S. cerevisiae* and closely related species in the *Saccharomyces sensu stricto* group. Interestingly, none of these non‐*S. cerevisiae* parental strains are naturally associated with the winemaking (or brewing) process because they are not as tolerant to high concentrations of sugar and ethanol as the *S. cerevisiae* parental strains (Borneman *et al*., 2012; Peris *et al*., 2012). These non‐*S. cerevisiae* parental strains display a complex aroma profile distinct from *S. cerevisiae*, produce low‐ethanol/high‐glycerol yields and are able to ferment at low temperatures, with the naturally occurring interspecific *Saccharomyces* hybrids generally exhibiting the desired fermentation characteristics of both parents (González *et al*., 2007).

Several studies have reported the superior properties of artificial interspecific hybrids for the winemaking industry. Researchers constructed and analysed *S. cerevisiae* × *Saccharomyces kudriavzeii* hybrids and evaluated the final product of laboratory‐scale fermentations of grape juice (González *et al*., 2007; Belloch *et al*., 2008). These researchers found that the hybrids had retained the high sugar and ethanol tolerance ability of its *S. cerevisiae* parent and displayed cryotolerance, along with the diverse aroma profile of the *S. kudriavzeii* parent. In one study, it was further shown that these hybrids produced intermediate concentrations of glycerol (at temperatures below 22°C) when compared to the parental strains, yielding a wine with a desired high‐glycerol, low‐ethanol content (González *et al*., 2007).

In another study, sparkling wines produced by two constructed *S. cerevisiae* × *S. uvarum* hybrids were analysed for sensory characteristics (Coloretti *et al*., 2006). Like the *S. kudriavzeii *× *S. cerevisiae* hybrids, these *S. cerevisiae *× *S. uvarum* hybrids also displayed properties from both parents, including increased glycerol production compared to the *S. cerevisiae* parent; however, no reduction of ethanol concentration was observed with other hybrids in the *sensu stricto* group (Coloretti *et al*., 2006). Unlike *S. kudriavzeii* and *S. uvarum,* which have been shown to be indirectly linked to the fermentation industry through *Saccharomyces* hybrid strains, benefits of incorporation of *Saccharomyces paradoxus* and *Saccharomyces mikatae* characteristics into wine strains were demonstrated in a recent study – diversifying the sensory composition of the wines and allowing tailoring wine aromas to satisfy different consumer requirements (Bellon *et al*., 2011, 2013, 2015). The *S. cerevisiae *× *S. mikatae* strain was particularly intriguing; it displayed heterosis (hybrid vigour) for enhanced tolerance to ethanol in relation to both parents. As with the other described hybrids, it produced less ethanol than its *S. cerevisiae* parent and about 20% more glycerol (Bellon *et al*., 2011). This demonstrates the potential of interspecific hybridization as a strategy to generate low‐ethanol wine strains.

## Directing the evolution of *Saccharomyces* strains towards glycerol and away from ethanol

Rational engineering strategies to redirect carbon flux from ethanol towards glycerol have provided great insight into potential biological mechanisms to lower alcohol content in wine. However, this approach is limited by two major problems. The first is that genetically modified (GM) food products continue to encounter resistance from consumers (Chambers and Pretorius, 2010), and the second is that the complexity of biological systems limits the power of rational engineering (Williams *et al*., 2016). An elegant solution to both of these problems is to use adaptive laboratory evolution to create strains with reduced ethanol yield via diversion of carbon flux towards glycerol (Dragosits and Mattanovich, 2013).

Adaptive laboratory evolution typically involves exposing a population of microorganisms to selective conditions such that the growth rate is significantly reduced. Over time, individual cells in the population will randomly accumulate mutations from DNA replication errors, and by chance some of these mutations will enable better growth under the selective conditions. Cells with advantageous mutations eventually take over the population to the point where the parental strain is no longer present. This process has been successfully employed in *S. cerevisiae* to achieve a variety of performance objectives such as heat tolerance (Caspeta *et al*., 2014), cold tolerance (López‐Malo *et al*., 2015), toxic compound resistance (Almario *et al*., 2013; Kildegaard *et al*., 2014; Brennan *et al*., 2015), altered wine yeast flavour profile (Cadière *et al*., 2012) and carbon source specificity (Wisselink *et al*., 2009; Garcia Sanchez *et al*., 2010; Zhou *et al*., 2012).

Most adaptive laboratory evolution experiments exploit the fact that the phenotype of interest is naturally coupled to cell survival. For example, simply by growing a population at a high temperature, any cell without a mutation for heat tolerance will die or be outcompeted by cells that do. However, this natural coupling does not normally occur for phenotypes such as metabolite overproduction. Creative solutions involving the use of metabolite responsive selective markers can be used to couple product yield to survival, although such mechanisms are not available for every metabolite of interest (Williams *et al*., 2016). In the case of adaptive laboratory evolution for glycerol production in *S. cerevisiae*, there is a potentially convenient solution to this problem. Glycerol acts as an osmoprotectant in yeast, and glycerol production can therefore be induced via the addition of salts to growth media to induce osmotic stress. This strategy was recently used with potassium chloride exposure to a wine yeast strain over 200 generations (Tilloy *et al*., 2014), resulting in a reduction in ethanol content of 1.3% (v/v) and a 41% increase in glycerol yield under non‐stress cultivation conditions. One potential concern with using osmotic stress to evolve glycerol production is the fact that acetic acid, acetaldehyde and acetoin are also overproduced during osmotic stress (Kutyna *et al*., 2010). Surprisingly, this was not the evolutionary outcome of the osmotic stress‐induced glycerol production phenotype, which suggested that central carbon flux had been altered via mutations in genes outside of the canonical high osmolarity glycerol (HOG) response pathway (Tilloy *et al*., 2014).

Glycerol production in *S. cerevisiae* can also be induced by the addition of sulfite to the growth medium (Petrovska *et al*., 1999), which binds to acetaldehyde to make it unavailable for ethanol production. This reduces glycolytic flux due to a shortage of NAD^+^ that would have been produced during ethanol fermentation, which can be restored by redirecting carbon through the NADH‐requiring glycerol synthesis pathway (Tilloy *et al*., 2015). Exposure of yeast to sulfite can therefore be used as a selection pressure for high‐glycerol production. This strategy was recently employed with great success, whereby exposure to sulfite over 300 generations resulted in a 46% increase in glycerol yield and a minor decrease in ethanol (Kutyna *et al*., 2012). Interestingly, when nine genes known to be involved in sulfite tolerance were sequenced in the evolved strain, none were found to be mutated. This result highlights the capacity of adaptive laboratory evolution to achieve engineering objectives via non‐intuitive mechanisms.

Adaptive evolution approaches are often time‐consuming primarily to do secondary mutations. These mutations can potentially affect key areas of yeast fermentation, for example fitness, and therefore reduce the ability of the evolved microbe to compete with other microorganisms during grape must. In addition to careful characterization and selection of mutants, crossing and back‐crossing to eliminate undesirable traits are usually required. The biggest attraction of adaptive evolution approaches for the wine industry, however, is that they do not involve genetic engineering and any strains obtained in this manner can be used immediately to produce commercial wine.

## Metabolic engineering of high‐glycerol, low‐ethanol *Saccharomyces* strains

**Figure 5:**
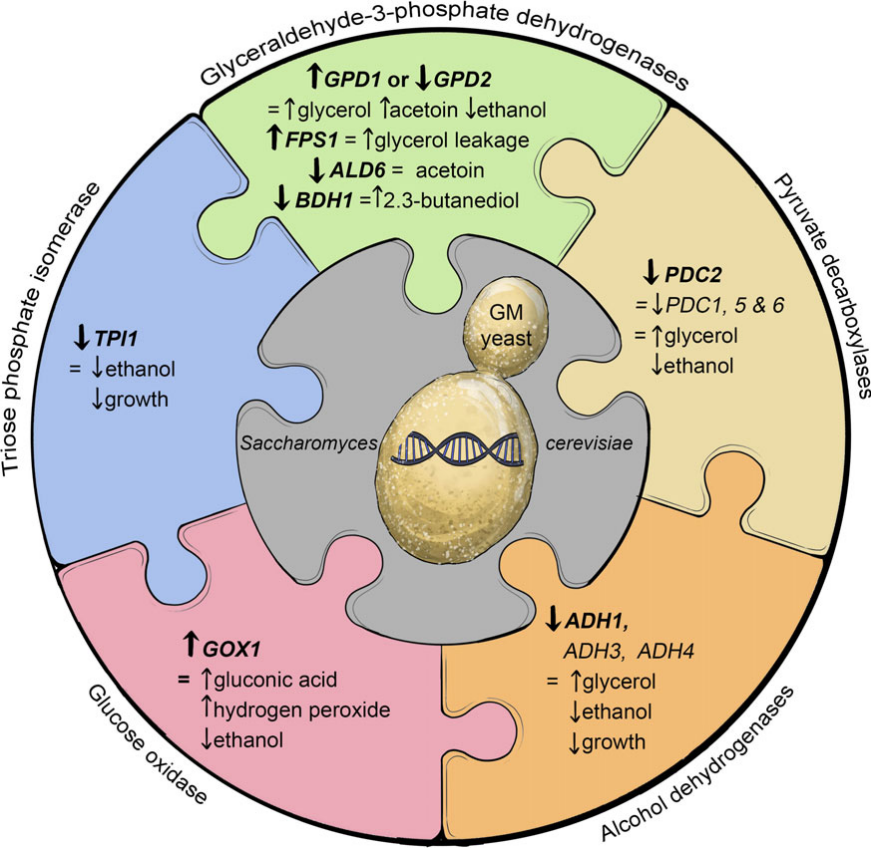
A schematic representation of genetic modification (GM) strategies by metabolic engineering to divert the metabolism of wine yeast away from ethanol formation by redirecting carbon to other end‐points such as glycerol. There are several strategies used to achieve this objective. These include (i) the overexpression of the yeast's own *GPD1* and/or *GPD2* genes, which encode glycerol‐3‐phosphate dehydrogenase isozymes; (ii) modification of the glycerol transporter encoded by *FPS1*; (iii) deletion of the *PDC2* gene encoding pyruvate decarboxylase; (iv) impairment of alcohol dehydrogenases encoded by *ADH1*,*ADH3*,*ADH4* and *ADH5*; (v) deletion of *TPI1*, which encodes triose phosphate isomerase. To ameliorate the formation of too much acetaldehyde (imparting ‘bruised apple’ notes), acetoin (imparting ‘rancid‐buttery’ notes) and acetic acid (imparting ‘vinegary’ notes) as a side‐effect of the overexpression of *GPD1* and/or *GPD2* in high‐glycerol/low‐ethanol yeast strains, the genes (*ALD1‐6*) encoding aldehyde dehydrogenases can be deleted.

**Figure 6:**
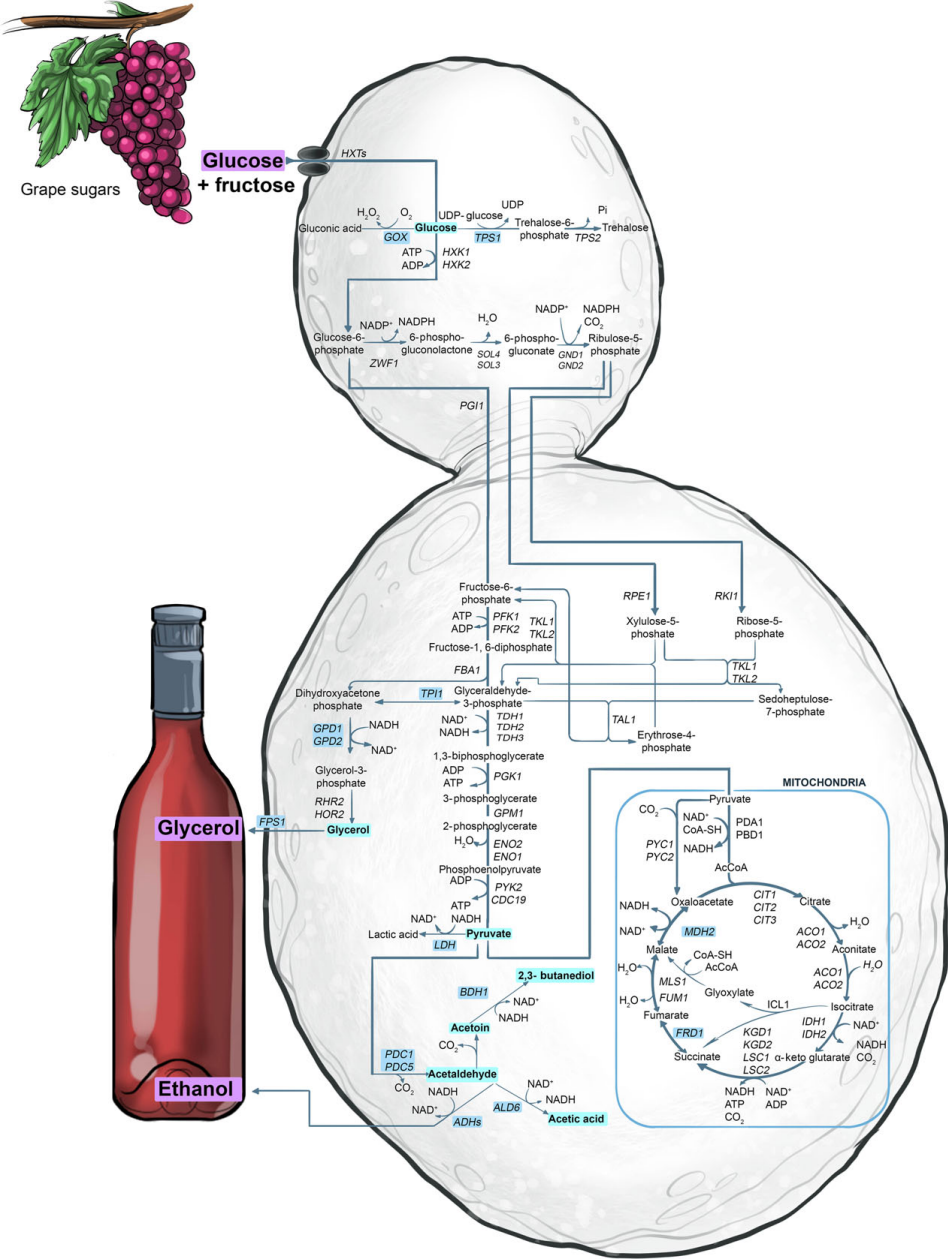
A schematic representation of carbon metabolism in wine yeast, including glycolysis, pentose phosphate pathway and tricarboxylic acid (TCA) cycle.

There are significant challenges relating to the anti‐GM constraints facing comestible products (reviewed by Pretorius, 2000). However, several genetic engineering strategies (Fig.**** 5) have been explored to generate wine yeasts that partially divert carbon metabolism away from ethanol production (Fig.**** 6), with the aim of decreasing ethanol yields during vinification (Cambon *et al*., 2006; Varela *et al*., 2012; Zhao *et al*., 2015). These attempts to generate low‐ethanol yeasts have been met with partial and mixed success (reviewed by Varela *et al*., 2012).

Some of the strategies included the expression of heterologous gene constructs in *S. cerevisiae*. These gene constructs encoded glucose oxidase (*GOX1*) from *A. niger* (Malherbe *et al*., 2003), the lactate dehydrogenase (*LDH*) from *Lactobacillus casei* (Dequin *et al*., 1999) and the H_2_O‐forming NADH oxidase gene (*nox*E) from *Lactococcus lactis* (Heux *et al*., 2006, 2008). The NAD^+^/NADH ratio was modified in *S. cerevisiae* strains carrying the latter, while strains that carried the *GOX1* and *LDH* gene constructs successfully redirected a portion of glucose to the production of gluconic acid and lactic acid. However, the inefficiency of glucose oxidase activity under anaerobic fermentative conditions and the sensory impact of lactic acid indicated that these two approaches did not prove to be viable solutions. Another approach involved extensive modification of *S. cerevisiae* hexose transporter genes (*HXT1*,* 2*,* 3*,* 4*,* 6* and *7*) aimed at forcing the GM yeast to respire rather than ferment regardless of the concentration of glucose and fructose it encounters in the culture medium (Henricsson *et al*., 2005). Restriction of sugar uptake by wine yeast cells would, however, result in stuck ferments and a failure to ferment grape must to dryness. Due to the mitigation of the Crabtree effect, this strategy also resulted in significantly low ethanol production, which would be unsuitable for winemaking.

Alternative strategies to generate low‐alcohol yeast strains targeted the *S. cerevisiae*'s endogenous *ADH* genes encoding alcohol dehydrogenases (Johansson and Sjostrom, 1984; Drewke *et al*., 1990), *TPI1* genes encoding triose phosphate isomerase (Compagno *et al*., 1996, 2001) and *PDC* genes encoding pyruvate decarboxylases (Nevoigt and Stahl, 1996). While some of these approaches were reasonably effective at redirecting carbon towards glycerol, the fermentation properties of these GM yeasts were unsuitable for winemaking. A further strategy to reduce ethanol yield during fermentation focussed on the diversion of carbon towards the synthesis of intermediates of the tricarboxylic acid (TCA) cycle. However, although the overexpression and deletion of several of the genes involved in the oxidative or reductive branches of the TCA cycle had an impact on the formation of organic acids, there was no effect on the production of ethanol (Varela *et al*., 2012). Another strategy aimed at lifting glucose repression from genes encoding enzymes involved in respiration. With this strategy, the idea was to channel carbon away from ethanol formation by deleting the *HXT2* and *MIG1* genes. These modifications resulted in insignificant decreases in ethanol yield (Varela *et al*., 2012).

So far, the strategy that generated the best potential for a viable – albeit not yet ideal from a wine sensory perspective – solution is the approach to channel a substantial portion of glucose to glycerol during glycolysis. Enhanced expression of either of the two paralogues, *GPD1* and *GPD2*, that code for *S. cerevisiae*'s two glycerol‐3‐phosphate dehydrogenase isozymes increased glycerol concentration by up to 548% (Nevoigt and Stahl, 1996; Remize *et al*., 1999; De Barros Lopes *et al*., 2000; Eglinton *et al*., 2002; Cambon *et al*., 2006). The expression of a truncated form of the *FPS1*‐encoded glycerol transporter in *S. cerevisiae*, which allows continuous glycerol leakage from the cell, has also been shown effective to increase glycerol production (Tamas *et al*., 1999).

In one study, two *S. cerevisiae* wine strains carrying several stable, chromosomally integrated *GPD1* gene constructs significantly reduced ethanol production in wine (Varela *et al*., 2012). These two GM wine yeasts were able to lower the ethanol content from 15.6% (v/v) to 13.2% (v/v) and 15.6% (v/v) to 12% (v/v) in Chardonnay and Cabernet Sauvignon wines respectively. Unfortunately, these two GM strains also produced unacceptable concentrations of acetaldehyde and acetoin, which negatively affect wine flavour.

## Striking a balance between ethanol and glycerol in wine is a matter of taste

As evident from several studies discussed in the previous sections, it is clear that glycerol is widely regarded as the key to the equation of how to produce low‐ethanol wine without impacting negatively on flavour (Swiegers *et al*., 2005; Ugliano and Henschke, 2009; Kutyna *et al*., 2012; Varela *et al*., 2012, 2015). However, it is also clear that this thirst for turning sunshine into well‐balanced wines demands a balancing act between the formation of glycerol and ethanol in yeast's metabolism.

In its purest form, this colourless polyol tastes slightly sweet, as well as somewhat ‘oily’ and ‘heavy’. At concentrations usually ranging between 5 and 12 g l^−1^ in table wine, glycerol has an apparent effect on the sweetness of wine. However, contrary to popular belief, glycerol makes only a very minor contribution to the apparent viscosity of wine and bears virtually no relation to the so‐called legs or tears left on the inside of a wine glass. Glycerol is known to impart sweetness at a threshold of about 5.2 g l^−1^ in white wine but more than 28 g l^−1^ would be needed to become noticeable in terms of viscosity and mouthfeel (Swiegers *et al*., 2005; Du *et al*., 2012).

In terms of the glycolytic pathway in *S. cerevisiae*'s fermentative metabolism, glycerol is the preferred metabolite to lure glucose away from ethanol formation. In terms of cellular ‘carbon budget’, glycerol is ‘expensive’ relative to complete oxidation of glucose to carbon dioxide and is therefore an effective sink for cellular carbon (Varela *et al*., 2012). However, with the overexpression of *GPD1* or *GPD2* in wine yeast, increased glycerol production was not only accompanied by a reduction of ethanol content but also by elevated concentration of undesirable metabolites, such as acetaldehyde, acetic acid, acetoin and 2,3‐butanediol (Fig.**** 6). This is due to a perturbation in the redox balance of the high‐glycerol/low‐ethanol engineered wine yeast. To restore the redox balance, the action of one or more of the five *ALD*‐encoded aldehyde dehydrogenase isozymes is required. These aldehyde dehydrogenases help maintain yeast's redox balance by reducing co‐enzymes NAD^+^ or NADP^+^, when they oxidize acetaldehyde to acetic acid and acetoin (Pretorius *et al*., 2012; Varela *et al*., 2012, 2015). At concentrations above their individual threshold values, acetaldehyde can make a wine smell ‘flat and vapid’ or elicit ‘bruised apple’ characters. Too much acetoin and acetic acid in wine can impart ‘rancid‐buttery’ and ‘vinegary’ notes respectively.

In a partially successful attempt to block the metabolic route towards acetaldehyde and acetic acid, wine strains were constructed in which the *ALD6* gene was deleted (Cambon *et al*., 2006; Varela *et al*., 2012). Acetic acid concentrations in wines made with such GM strains were within the range considered acceptable for high‐quality wines. However, the concentration of acetaldehyde was above the sensory threshold and elicited an undesirable ‘bruised apple’ smell in wines (Eglinton *et al*., 2002; Cambon *et al*., 2006; Varela *et al*., 2012). To address this issue, the *BDH1* gene encoding butanediol dehydrogenase was overexpressed to divert the carbon flux from acetaldehyde away from acetic acid towards acetoin and the sensorially neutral metabolite 2,3‐butanediol. This resulted in a significant decrease in the formation of acetaldehyde, acetic acid and acetoin; however, unexpectedly the overexpression of *BDH1* also altered the production of glycerol and ethanol, most likely driven by changes in redox balance (Varela *et al*., 2012).

In summary, although valuable information has been unearthed with these exploratory research programmes so far, the development of ‘winery‐ready’ low‐alcohol yeast is still very much a work‐in‐progress. It has become clear that trying to produce better ‘balanced’ fruity wines from well‐ripened grapes is much more complex than initially assumed. By increasing the formation of glycerol at the expense of ethanol during fermentation, the redox balance in the metabolism of yeast cells is upset and that results in high‐glycerol/low‐ethanol wine with unacceptable concentrations of other metabolites that have an unfavourable impact on the overall sensory quality of the wine.

Is there a way around this ‘brick wall’ with another renaissance in genetic techniques? At what point do we stop trying to use the same approaches? We know that the light bulb was not invented by continuously improving the candle – so, is there a better way?

## A call for new thinking and a fresh approach

There is no question that the global industry has a real need to provide consumers with ‘balanced’ wines containing lower concentration of alcohol without compromising the highly desirable ripe fruit flavours from well‐matured grapes. And if it is true that ‘necessity is the mother of invention’, then it is time for thinking outside the square of last‐century technologies. In the context of development of low‐alcohol yeast, the time is ripe to explore the potential of the new emerging science of synthetic biology and potentially ‘game‐changing’ technologies, such as synthetic genomics and DNA editing techniques. No discovery of the past century holds more promise – or raises more troubling ethical questions – than synthetic biology.

For an industry steeped in tradition, it might well be a frightening thought that a large international project – the Synthetic Yeast Genome (Sc2.0) Project – is on track to synthesize all 16 chromosomes of a laboratory strain of *S. cerevisiae* and deliver the world's first eukaryote with a chemically synthesized genome by 2018 (recently reviewed by Pretorius, 2016).

Synthetic biology and CRISPR‐Cas9 DNA editing technologies have also been applied to convert yeast into ‘cell factories’ for the production of low‐volume/high‐value compounds, such as (i) artemisinic acid (a precursor of the potent antimalarial compound, artemisinin); (ii) resveratrol (the antioxidant found in, amongst others, red wine and believed by some to be associated with anti‐ageing, antidiabetic, anti‐inflammatory, antithrombotic and antitumour properties); (iii) vanillin (the most widely used flavouring agent); (iv) stevia (a zero‐calorie sweetener); and (v) saffron (the world's most expensive spice) (Pretorius, 2016).

It might even be more unsettling for some wine industry stakeholders if they learn that the future of a ‘wine yeast 2.0’ is already here. To demonstrate the transformative power of synthetic biology, a wine yeast strain (AWRI1631) containing a set of chemically synthesized, codon‐optimized genes was recently constructed to produce Chardonnay wine that smells and tastes like raspberries (Lee *et al*., 2016). The following genes were synthesized and successfully expressed in this wine strain for the production of the raspberry ketone, 4‐[4‐hydroxyphenyl]butane‐2‐one: *RtPAL* from an oleaginous yeast, *Rhodosporidium toruloides*;* AtC4H* from the well‐studied model plant, *Arabidopsis thaliana*;* Pc4CL2* from parsley, *Petroselinum crispum*; and *RpBAS* from rhubarb, *Rheum palmatum*.

It is therefore not beyond the realms of possibility to envision that similar synthetic biology‐based strategies will be successfully harnessed for the development of low‐alcohol/high‐glycerol wine yeasts with flavour‐enhancing capabilities. But what are the implications of genome engineering as opposed to genetic engineering? We know all too well that, while genetically engineered medicine has been accepted widely, food products fashioned in similar ways have not, despite the scores of studies demonstrating that such products (e.g. bioengineered yeast‐derived chymosin in cheese manufacturing) are no more unsafe to eat than any other food. As the furore over the labelling of GM food products has demonstrated time and time again, it does not matter whether a product is safe if people refuse to consume it. It is hoped that synthetic biology tools, such as CRISPR DNA editing technologies, might provide a way out of this scientific and cultural quagmire. CRISPR technologies provide researchers with the ability to redesign specific genes and gene networks without having to introduce DNA from other organisms. In some countries, such as Argentina, Germany and Sweden, regulators have already made a distinction between genetically modified organisms (GMOs) and organisms edited with CRISPR technologies. There are also strong indications that the US Food and Drug Administration might follow suit. This could make CRISPR‐designed products more readily available and easily regulated than any other form of GM drug or food. Whether the public will take advantage of them remains to be seen.
